# Editorial: Stress echocardiography in cardiovascular diseases

**DOI:** 10.3389/fcvm.2024.1407374

**Published:** 2024-05-27

**Authors:** Lixue Yin

**Affiliations:** Sichuan Academy of Medical Sciences and Sichuan Provincial People’s Hospital, Chengdu, China

**Keywords:** stress echocardiography, coronary artery disease, valvular heart disease, adverse events, hypertrophic cardiomyopathy

Editorial on the Research Topic Stress echocardiography in cardiovascular diseases

Stress echocardiography has gained widespread acceptance globally, with its clinical utilization increasing annually. The procedure involves the use of echocardiography, which is a type of ultrasound imaging, to assess the heart's structure and function during periods of stress, such as physical exercise or the administration of pharmacological agents. This allows doctors to detect abnormalities that might not be apparent during rest. This diagnostic modality offers a comprehensive array of systematic diagnostical information, ranging from etiology to pathophysiological mechanisms, thereby enhancing the clinical understanding and diagnostic capabilities of non-invasive imaging (1). It also improves the decision-making process for treating various cardiovascular diseases. Multi-center studies have consistently demonstrated that stress echocardiography exhibits a higher specificity, sensitivity, and accuracy exceeding 90% in the diagnosis of ischemic heart disease (2). This high diagnostic accuracy makes stress echocardiography an invaluable tool in the evaluation of patients with suspected cardiovascular disease. With the advancement of related technology and research, it is expected that stress echocardiography will continue to play a pivotal role in cardiovascular diagnostics, leading to better patient's outcomes and improved quality of life.

Stress echocardiography, introduced in the 1970s for cardiovascular disease assessment, remains underutilized in developing countries. Key factors limiting its clinical application are the absence of conclusive evidence on its efficacy across diverse racial groups, resulting in reluctance among cardiologists to adopt it. Additionally, the technique's widespread implementation is hindered by a scarcity of experienced cardiologists and sonographers capable of consistently acquiring and analyzing images during stress testing. Furthermore, challenges arise from the selective use of stress tests tailored to the diagnostic requirements of various diseases at different stages. Currently, there is no established system of targeted and reliable observation parameters tailored to distinct observation goals, necessitating large-scale cohort studies for validation. Ultimately, a profound comprehension of the pathophysiology of cardiovascular diseases across genders, ages, and disease types and severities under varying stress conditions remains elusive (1, 3–5).

Building upon these obstructions, the cardiologists and physiologists of Europe, the United States and China have embarked on the 2030 stress echocardiography research plan, aiming to further expand the scope and depth of its clinical applications (4).

For a long time, the application of traditional stress echocardiography has focused on ischemic heart disease. However, over the past 10 years, the use of stress echocardiography has exploded systematically. From the application of gray-scale and single modality imaging (M-mode and two-dimensional echocardiography to evaluate the cardiac ventricular wall motion of patients with known or suspected coronary artery disease) to the current integration of multi-modality technical methods (from M-mode ultrasound to two-dimensional, pulsed, continuous, color and tissue Doppler ultrasound, lung ultrasound, real-time three-dimensional echocardiography, speckle tracking and myocardial perfusion imaging), the relevant technical methods have gradually covered various cardiovascular disease spectrum (form coronary artery disease, to valvular disease, heart failure, cardiomyopathy, pulmonary hypertension and neoplastic heart disease, etc.) and all age groups (from children with congenital heart disease and tumors to the elderly with various degenerative heart diseases). Based on the cardiovascular stress center, a variety of stress modes could be used to provide more targeted information on cardiovascular morphological stress status for cardiovascular structure, myocardial and valvular function, myocardial perfusion, fluid-structure interaction and cardiovascular coupling according to different clinical diagnostic goals (Hirasawa et al., Duan et al., Li et al., Lee et al., Su et al.).

In the field of routine cardiovascular practice, the integrated stress echocardiography concept holds immense value for achieving a comprehensive and systematic understanding of the emergence and progression of various diseases (1, 3, 4).

For this stress echocardiography topic, these published papers revealed some clinical potentials: There is evidence indicating that patients experiencing angina with no obstructive coronary artery disease (ANOCA) exhibit coronary microvascular dysfunction (CMD), which can be assessed noninvasively using myocardial work indices (MWIs) and the left ventricular pressure-strain loop (LV PSL) (Li et al.). This abnormal increased MWIs combined with LV PSL might suggest a novel approach for detecting LV systolic function noninvasively among ANOCA patients with CMD. Hirasawa et al. reviewed recent advancements in the management of valvular heart disease (VHD) through stress echocardiography, emphasizing its importance in accurately assessing disease severity and guiding decisions on transcatheter interventions or open heart surgery. Current evidence underscores the crucial role of stress echocardiography in determining interventional indications and risk stratification in mitral regurgitation and aortic stenosis Hirasawa et al., Lee et al. examined the safety profile of stress echocardiography, noting that while this test is associated with a low overall prevalence of severe, life-threatening adverse events, dobutamine stress echocardiography may have a higher risk of adverse events, even during the COVID-19 pandemic. Su et al. aimed to evaluate left atrial strain (LAS) in patients with hypertrophic cardiomyopathy (HCM) using treadmill exercise stress echocardiography, incorporating three-dimensional speckle tracking technology. Their findings hints that left atrial reservoir strain (LASr) exhibits the strongest association with METS ≤ 6.0, and rest-LASr is the most reliable predictor of METS ≤ 6.0 for distinguishing various HCM subtypes. This discovery indicates that rest-LASr assessment could be a dependable method to assess the functional status of HCM patients unable to undergo exercise testing Su et al.

These published papers bring together the valuable clinical practice and diagnostic experience accumulated by colleagues from the hospitals all over the world. The papers try to explain in detail the basic principles, technical methods and clinical applications of stress echocardiography for cardiovascular physicians, cardiovascular surgeons, cardiovascular sonographers and related professional staff through concise, easy-to-understand language, detailed images and data analysis. It is hoped that the publication of these papers will help us to promote the extensive and in-depth clinical application of stress echocardiography in the world and more effectively improve the diagnosis and treatment of cardiovascular diseases.
